# Effects of Stress Exposure to Pain Perception in Pre-Clinical Studies: Focus on the Nociceptin/Orphanin FQ–NOP Receptor System

**DOI:** 10.3390/brainsci14090936

**Published:** 2024-09-19

**Authors:** Pietro Pola, Alessia Frezza, Elaine C. Gavioli, Girolamo Calò, Chiara Ruzza

**Affiliations:** 1 Department of Neuroscience and Rehabilitation, University of Ferrara, 44121 Ferrara, Italy; pietro.pola@unife.it (P.P.); alessia.frezza@unife.it (A.F.); 2 Department of Biophysics and Pharmacology, Federal University of Rio Grande do Norte, Natal 59078-900, Brazil; elaine.gavioli@ufrn.br; 3 Department of Pharmaceutical and Pharmacological Sciences, University of Padua, 35131 Padua, Italy; girolamo.calo@unipd.it; 4 LTTA Laboratory for Advanced Therapies, Technopole of Ferrara, 44121 Ferrara, Italy

**Keywords:** nociceptin/orphanin FQ, NOP receptor, stress, pain, animal models

## Abstract

Exposure to physical and psychological stress modulates pain transmission in a dual manner. Stress-induced analgesia (SIA) refers to the reduction in pain sensitivity that can occur in response to acute stress. On the contrary, chronic stress exposure may lead to a phenomenon named stress-induced hyperalgesia (SIH). SIH is a clinically relevant phenomenon since it has been well documented that physical and psychological stress exacerbates pain in patients with several chronic pain syndromes, including migraine. The availability of animal models of SIA and SIH is of high importance for understanding the biological mechanisms leading to these phenomena and for the identification of pharmacological targets useful to alleviate the burden of stress-exacerbated chronic pain. Among these targets, the nociceptin/orphanin FQ (N/OFQ)–N/OFQ peptide (NOP) receptor system has been identified as a key modulator of both pain transmission and stress susceptibility. This review describes first the experimental approaches to induce SIA and SIH in rodents. The second part of the manuscript summarizes the scientific evidence that suggests the N/OFQ–NOP receptor system as a player in the stress–pain interaction and candidates NOP antagonists as useful drugs to mitigate the detrimental effects of stress exposure on pain perception.

## 1. Introduction

Exposure to physical and psychological stress modulates pain transmission in a dual manner. Stress-induced analgesia (SIA) refers to the reduction in pain sensitivity that can occur in response to acute stress. This adaptive response allows an organism to maintain functionality and respond to threats despite potential injury or pain. SIA is mainly due to the release of endogenous opioids and endocannabinoids during acute stress experience [1]. On the contrary, stress-induced hyperalgesia (SIH) is an increased sensitivity to pain in response to chronic stress [2]. SIH is a clinically relevant phenomenon since it has been well documented that physical and psychological stress exacerbates pain in patients with chronic pain syndromes, i.e., fibromyalgia [3,4,5,6], inflammatory bowel diseases [7], complex regional pain syndrome [8,9], and shoulder/neck pain syndrome [10,11]. Stress may also represent a migraine trigger since it reduces the threshold of a migraine attack in susceptible patients [12,13]. The clinical relevance of SIH underlines the importance of stress management in pain pathologies. The mechanisms by which stress worsens pain are complex and only partially understood; they involve, at least in part, the activation of the hypothalamic-pituitary-adrenal (HPA) axis with increased cortisol levels, the dysregulation of monoaminergic systems, as well as central sensitization and neuroinflammation [2]. The identification of those biological systems that play a role in the interplay between stress and nociception and their pharmacological modulation is crucial for the development of novel strategies to alleviate the burden of chronic pain. To this end, the availability of SIA and SIH animal models is of paramount importance. The present narrative review describes the experimental approaches to induce SIA and SIH in rodents. The second part of the manuscript summarizes the scientific evidence that suggests the nociceptin/orphanin FQ (N/OFQ)–N/OFQ peptide (NOP) receptor system as a player in the stress–pain interaction and proposes NOP antagonists as drugs that may be useful to mitigate the detrimental effects of stress exposure on pain perception.

## 2. Animal Models of Stress-Induced Analgesia

Acute stressors have been strongly validated as analgesia inductors. In the scientific literature, there are numerous examples of animal models aimed at mimicking SIA, including unconditioned SIA and conditioned SIA (also known as fear-conditioned analgesia). Unconditioned SIA arises with an unconditioned aversive stimulus or environment that is capable of inducing analgesia, whereas conditioned SIA happens due to the re-exposure to a context that has been previously associated with a noxious or aversive stimulus [1]. Of note, the study of the biological mechanisms involved in SIA led to a better understanding of those endogenous systems able to suppress pain, which is useful to individuate new therapeutic targets for the treatment of pain-related disorders. In rodents, the effect of acute stress on pain perception has been investigated both using classical nociceptive assays (i.e., tail flick and withdrawal [14,15,16], hot plate [17,18,19], and tail pinch [20,21]) and assays mimicking inflammatory/chronic pain (i.e., formalin test [22,23,24]). Many environmental stimuli have been demonstrated to produce analgesia. Importantly, the use of different stimuli and different experimental conditions may lead to different results due to the activation of different biological substrates evoking analgesia. Most studies performed footshock, forced swimming, or restraint stress, among others.

### 2.1. Footshock Stress

Footshock stress has been used to induce SIA mostly in rats, but some studies have been performed on mice as well, and the resulting analgesia has been defined as footshock-induced analgesia (FSIA). The electric foot shock paradigm includes acute exposures of shocks of varying intensity and duration (3–30 min) on an electrified grid floor. Three min of footshock induced analgesia in an intensity-dependent manner, with robust SIA levels reaching from 0.6 mA [25]. Some studies have hypothesized that the duration of acute footshock stress influences the nature of resulting analgesia, with brief and continuous shocks (3 min) leading to analgesia resistant to the opioid antagonist naloxone and prolonged and intermittent shocks (30 min) leading to naloxone-reverted analgesia in rats [26,27,28]. Other studies stated that the nature of SIA induced by footshock depends on the body region shocked. In particular, front paw shocks produce a naloxone-reversible analgesia, and hind paw shocks produce an analgesia that fails to be attenuated by the opioid receptor antagonist in rats [29,30]. In general, in opioid-dependent SIA, opioid receptors have been reported to be involved both at spinal and supra-spinal levels. Otherwise, the endogenous cannabinoid system has also been identified as an endogenous system activated by electric footshock and important for SIA [25,31,32].

### 2.2. Forced Swim Stress

The forced swim stress (3–6 min) is another widely used method to induce SIA both in rats and mice. It has been reported that SIA induced by a few min of swimming appeared immediately after stress, lasted less than one hour, and was subjected to tolerance [33]. Swim stress-induced analgesia (SSIA) has been classically measured using acute nociceptive assays (i.e., hot plate, tail flick, and tail withdrawal assays), but acute swim is also able to reduce the nociceptive effects of formalin, both in the first and second phases [34,35,36,37]. One of the experimental conditions that can influence the nature of SSIA is water temperature. Specifically, cold water (20–15 °C) swimming produces non-opioid SSIA in rats and mice [38,39,40], whereas warm water (32 °C) swimming has been reported to produce SSIA blocked by the short-acting opioid antagonist naloxone or by the long-acting opioid antagonist naltrexone [41,42,43,44], thus an opioid-dependent SSIA. Focusing on the endogenous opioids involved in SSIA, a 2011 study found that antinociception induced by forced swimming (32 °C) was attenuated in beta-endorphin-deficient mice but not in mice lacking enkephalins or dynorphins, suggesting that beta-endorphin is the primary neural substrate mediating this form of SIA [45]. Sex-specific differences in the neurochemical mediation of SSIA have been reported [46]. In this study, non-opioid SSIA after 15 °C swim was attenuated by the N-methyl-D-aspartate (NMDA) receptor antagonist dizocilpine in male but not female mice. Moreover, ovariectomized female mice were sensitive to dizocilpine antagonism of non-opioid SSIA. This finding suggests the existence of an estrogen-mediated mechanism of SSIA. Interestingly, SSIA has been the model used to determine the role played by the orexin system in SIA. It has been reported that orexin receptor 1 and orexin receptor 2 are both important in non-opioid SIA [34,47]. More in detail, the brain areas in which the activation of orexin receptors 1 and 2 seems important for SIA are the nucleus accumbens [24,48] and the dentate gyrus of the hippocampus [49,50].

### 2.3. Restraint Stress

Restraint stress consists in placing the animal (mouse or rat) in a well-ventilated tube or small cage to severely restrict its movements for a period from 30 min to 3 h. Usually, a single exposure to this stressor is sufficient to induce SIA [15,51,52,53,54,55], while, as described below, repeated sessions are used to induce SIH. Similar to forced swim stress, restraint SIA can be measured using both an acute nociceptive test [56,57,58] and the formalin test [59,60,61]. The exact mechanisms by which restraint evokes pain suppression are not known, although an involvement of opioid mechanisms has been demonstrated since restraint-induced analgesia was prevented by naltrexone [42]. More recent studies have also revealed orexin involvement [58,60,61,62,63,64] and orexins—N/OFQ interaction [56].

## 3. Animal Models of Stress-Induced Hyperalgesia

Starting in the ‘90s, several pre-clinical studies have been performed to develop SIH animal models. Different stressors have been used as well as different ways to assess hyperalgesia. Similar to SIA, the use of stressful procedures different for their nature, intensity, and duration may lead to different results since different biological pathways may be activated under different experimental conditions. In general, to obtain SIH, a long-lasting or repeated stressful stimulus should be applied. The most commonly used stressors are forced swimming, restraint, and repeated cold stress. More rarely, in some studies, chronic mild stress, early-life stress, water avoidance, as well as other kinds of stress have been used [2].

### 3.1. Forced Swim Stress

Forced swimming to induce SIH has been used primarily in rats, where a 3-day protocol (one swim session/day, 10–20 min, 24–26 °C) is sufficient to induce per se thermal and mechanical hyperalgesia, starting one day after the last stress section and lasting up to one week [65,66,67,68,69]. The same procedure was able to increase the nociceptive effect of formalin, especially during the second inflammatory phase [66,70,71,72], of the Complete Freund’s adjuvant (CFA) [73,74,75,76,77], and of carrageenan [65], suggesting that forced swimming stress may worsen inflammatory pain. Of note, some research groups reported that 3 days of forced swimming failed to induce SIH in rats [78] and longer protocols (from 7 to 14 days) must be eventually adopted [78,79,80]. Little is known regarding the biological mechanisms leading to SIH after swim stress; however, increased inflammation [68,69] and reactive oxygen species (ROS) levels [73], together with an unbalance of the GABAergic/glutamatergic transmission [71,72] in the spinal cord, have been reported. The treatment with classical antidepressants [66,80], valproate [67], and nonsteroidal anti-inflammatory drugs (NSAIDs) [68] was able to prevent/treat the SIH induced by forced swimming.

### 3.2. Restraint Stress

Restraint stress has been largely used to induce SIH both in mice and in rats. In rodents, the effects of a single session of restraint, lasting from one to three hours, elicited variable effects in different laboratories, ranging from analgesia [51,81,82], no effect [83,84,85], and hyperalgesia [85]. On the contrary, the use of multiple restraint sessions to produce SIH elicited consistent results among different laboratories. Commonly, mice or rats are placed in the restrainer once a day for a period of 1 to 6 h, for 7 to 40 days. Similar protocols are used for mice and rats. The chronic restraint stress leads to mechanical [84,86,87,88,89,90,91,92,93,94,95,96,97] and thermal [84,90,92,94,97] allodynia and to thermal hyperalgesia [51,84,86,87,95,96,97,98,99,100], that can be measured using the von Frey filaments, the hot plate and tail flick/withdrawal assays, and the paw withdrawal to cold. Mechanical allodynia applies not only to the paw but also to the masseter muscle [101,102,103], indicating an effect also in the trigeminal area. The increased nociceptive behavior is already evident after few restraint sessions (i.e., 3–7 days) and, after 28 or 40 days of stress, almost 30 days without stress are required to restore the basal conditions [88,90,93]. Thus, chronic restraint stress precipitates a long-lasting increased pain perception. Similar to swim stress, restraint stress was also able to increase the nociceptive response of mice and rats to formalin [83,86,90,104], prostaglandin E_2_ (PGE_2_) [105], and bee venom [106], to worsen the neuropathic pain induced by infraorbital nerve chronic constriction injury [107] and the pain induced by nerve growth factor injection into the low back muscles of mice [108]. Interestingly enough, in a mouse endometriosis model, chronic restraint stress increased nociception and exacerbated the course of the disease [109]. Collectively, the large number of studies performed using chronic restraint stress as a model of stress demonstrated that this procedure triggers SIH in a robust manner, leading to similar effects in mice and rats, with reproducible data among different laboratories. As forced swim, restraint stress is associated with increased cortisol levels [83,88,110], as well as inflammation markers in the spinal cord [88,93,95,105] and in the trigeminal nucleus caudalis and ganglion [101,102,103]. The blockage of the glucocorticoid receptor as well as of some inflammatory pathways (i.e., interleukin-1 (IL-1) receptor, toll-like receptor 4 (TLR4), and high mobility group box 1 (HMGB1) protein; cyclooxygenase-2 (COX2), and E-type prostanoid receptor 4 (EP4)) counteracts SIH [93,94,105,110]. Additionally, SIH evoked by restraint is counteracted by the classical antidepressant acting as a selective serotonin reuptake inhibitor (SSRI), fluoxetine [96]. Chronic restraint stress has been used in different studies to investigate the role played by the opioid system in SIH. These studies revealed that prolonged stress exposure leads to plastic changes of the opioid system, mainly regarding the mu receptor. Specifically, a reduction in the density of opioid receptors, evaluated by binding experiments, has been detected in the spinal cord, cortex, and hippocampus of rats subjected to chronic restraint stress [111], as well as a reduction of morphine sensitivity [83,90,104]. Both findings have been confirmed using different stress models [112,113,114,115]. Thus, chronic stress may cause a down-regulation of endogenous opioidergic pathways, and this phenomenon may, at least in part, contribute to the development of SIH. For a review on the role of the opioid system in SIA and SIH, see [116].

### 3.3. Repeated Cold Stress

Repeated cold stress consists in placing mice or rats in a cold room (usually 4 °C) for several hours/day for different days. Typically, after being kept overnight at 4 °C, animals are alternately exposed to room temperature and cold temperature at 30 min intervals from 10:00 AM to 5:30 PM, followed by another overnight period at 4 °C. This is repeated for 3 to 5 days. Repeated cold stress induces mechanical and thermal hyperalgesia [117,118,119,120,121,122,123,124,125,126], and, because of the induction of persistent muscle pain measured with the Randall–Selitto test, it has been proposed by some authors as a fibromyalgia model [127].

From a pharmacological point of view, it is worth noting that drugs able to increase the synaptic levels of serotonin and norepinephrine (i.e., fluoxetine, clomipramine, 5-hydroxytryptophan, and milnacipran) are able to reverse SIH induced with different procedures [66,70,80,83,128]. This suggests that monoamine dysfunction may be involved in SIH and that their potentiation may represent a therapeutic approach. This is not surprising considering the role played by monoamines in descending pain pathways and the efficacy of antidepressant drugs for the management of neuropathic pain [129].

## 4. Animal Models of Stress-Induced Migraine

Migraine is a multifactorial and common disorder that affects millions of people worldwide. Although recognizing a migraine trigger can be difficult, several patients reported stress as the primary trigger for migraine attacks [130]. Over the years, several pre-clinical migraine models have been set up and validated. These models helped to better understand the biological bases of the disease and are useful for the identification of new therapeutic opportunities. To mimic migraine in mice and rats, several stimuli have been used, ranging from the electrical stimulation of the trigeminal nerve or ganglion to the local or systemic injection of chemical stimuli. The administration of nitroglycerin (GTN), calcitonin gene-related peptide (CGRP), and transient receptor potential cation channel subfamily A member 1 (TRPA1) agonist is the most widely used approach to induce migraine-like pain in rodents, commonly measured as mechanical allodynia in the peri-orbital area or in the paw [131,132]. To elucidate the complex interplay between stress and migraine, recent studies have employed a paradigm where stress is induced prior to exposure to a known migraine trigger. Among these investigations, it is important to distinguish between those where stress precedes a fully effective stimulus, capable per se of inducing migraine, and those where stress precedes a stimulus that is not active per se (i.e., a subthreshold dose of a migraine stimulus). In the first case, chronic stress (restraint [133], chronic unpredictable, and social defeat [134] stress) did not change the pro-allodynic effects of an active dose (10 mg/kg) of GTN. Similarly, chronic variable stress did not affect cortical spreading depolarization in a genetic mouse model of migraine (mice with the familial hemiplegic migraine type 1 mutation, FHM1) [135]. Of note, quite a different result has been reported by Raoof et al. demonstrating that the exposition of rats to chronic unpredictable stress or to maternal separation exacerbates the effects of GTN 5 mg/kg, only in female animals [136]. It can be hypothesized that, in those studies using a fully effective trigger, a ceiling effect that prevents to appreciate the worsening effect of stress on migraine-like pain may occur. In fact, when chronic stress is applied before a sub-threshold migraine inducer, a clear effect of stress has been reported by different research groups. Avona et al. demonstrated that 2 h of restraint stress for 3 consecutive days induce periorbital mechanical allodynia that disappears 14 days after stress. More interestingly, after returning to baseline, a subthreshold dose of the nitric oxide donor sodium nitroprusside (0.1 mg/kg) was effective in stressed but not naïve mice [137]. The authors described this condition as “latent sensitization”, wherein initial stress increases the animal sensitivity to the migraine trigger. A similar pattern of effects has been replicated using the TRPA1 agonist umbellulone [138] and the pituitary adenylate cyclase-activating polypeptide-38 (PACAP38) [139] as chemical inducers. Of note, repeated unpredictable sound stress has also been reported to induce per se migraine-like signs; however, its ability to prime a subthreshold migraine trigger has not been investigated [140]. Of interest, in this last model, female animals were more sensitive than male animals. As far as the effectiveness of standard antimigraine drugs in this model, different results are reported. The study by Avona et al. described the inactivity of sumatriptan in reverting sodium nitroprusside effects, while a CGRP antibody was fully active [137]. Another study reported that the beta blocker propranolol and the CGRP receptor antagonist olcegepant are able to prevent allodynia and priming when given before stress exposure, while sumatriptan reverted the effects of umbellulone [138]. These findings are in line with the clinical use of these drugs; in fact, beta-blockers and CGRP drugs are approved for migraine prophylaxis, while sumatriptan is for migraine attack abortion. The pathophysiological aspects of the stress–migraine correlation are still poorly understood. Studies suggest that stress exposure leads to the release of PACAP38 [139] and, in females, of prolactin [141] and that these peptides are both important for the priming effect of stress on migraine-trigger susceptibility. Additionally, the mitogen-activated protein kinase interacting protein kinases (MNK) pathway has also been reported to be important in a stress-induced migraine model [142]. Interestingly, the kappa opioid receptor antagonist norbinaltorphimine (nor-BNI) given before stress exposure prevented the deleterious effect of stress on migraine susceptibility, suggesting the kappa receptor as a pharmacological target for the prevention of migraine triggered by stress [138].

Table 1 summarizes the effects of the most commonly used stress procedures on pain perception. The table highlights the species used in the studies (rat vs. mouse) as well as the sex of the animals. Any sex-related differences are noted in the comments.

## 5. The N/OFQ–NOP Receptor System

Nociceptin/orphanin FQ (N/OFQ) is the endogenous ligand of a G-protein-coupled receptor now named NOP receptor [143]. The N/OFQ–NOP system is the first successful example of reverse pharmacology [144,145]. A G-protein-coupled receptor structurally similar to opioid receptors was cloned and named opioid receptor-like 1 (ORL-1) [146]. One year later, two distinct research groups identified an endogenous 17-aminoacid neuropeptide that binds with high affinity to the ORL-1 [147,148]. This peptide was named by the Meunier’s group nociceptin due to its ability to elicit nociceptive effects after intracerebroventricular (i.c.v.) administration in mice [147]. Reinscheid and colleagues named the newly discovered peptide orphanin FQ to indicate a ligand of an orphan receptor with phenylalanine (F) and glutamine (Q) at the N- and C-terminals, respectively [148]. Despite structural homology with classical opioid systems, neither the endogenous opioids display affinity for the NOP receptor nor does N/OFQ bind opioid receptors [149]. The NOP receptor, when activated, inhibits the formation of cyclic adenosine monophosphate (cAMP), closes voltage-gated Ca^2+^ channels, and opens inwardly rectifying K^+^ channels [150,151,152,153]. Thus, NOP receptor activation reduces neuronal excitability and neurotransmitter release. NOP receptor activation has been shown to reduce the release of several neurotransmitters, including norepinephrine, dopamine, serotonin, acetylcholine, and glutamate. The reduction of neurotransmitter release is the mechanism by which the N/OFQ–NOP receptor system modulates many biological functions [154]. Since its discovery, numerous studies have investigated the role of the N/OFQ–NOP system in nociception, revealing a complex picture. In rodents, the effects of NOP agonists on pain transmission range from pro-nociceptive to analgesic effects, depending on the site of action (supraspinal, spinal, peripheral) and on the animal model used [155,156,157]. N/OFQ and NOP agonists produce pro-nociceptive effects when given supraspinally (i.c.v.) in mice and rats in models of acute pain [147,149,158,159]. This action is likely due to the presence of NOP receptors on the OFF cells in the rostral ventromedial medulla. These neurons evoke a “descending inhibition” of the afferent neurons from the spinal cord. NOP activation directly inhibits OFF cell firing, thus producing hyperalgesia [154,156]. Differently, when NOP agonists are injected intrathecally, a robust analgesic effect has been recorded in acute pain tests [160,161,162,163,164] and in chronic pain models [165,166,167,168,169]. In the spinal cord, NOP agonism produces analgesia by inhibiting excitatory glutamatergic nociceptive transmission both at the pre- and postsynaptic levels. Activation of presynaptic NOP receptors inhibits voltage-gated Ca^2+^ channels, leading to a reduction of glutamate release from primary afferent fibers. Additionally, activation of postsynaptic NOP receptors, opening K^+^ channels, hyperpolarizes and hence inhibits electrical activity of secondary afferent neurons [154,156,170]. Interestingly enough, N/OFQ spinally administered was more effective as analgesic in chronic than in acute pain models, but the neurobiological mechanisms to explain this phenomenon are still not understood [157]. Finally, non-peptide NOP agonists given systemically resulted inactive in acute nociception [171,172] but efficacious in reducing chronic and inflammatory pain [173,174,175,176,177]. Interestingly, the ability of a systemic NOP agonist to counteract pain in a mouse model of migraine induced by GTN has been recently described [178]. In addition to the numerous studies that have linked the NOP receptor to pain, a large amount of scientific literature also demonstrated the involvement of this receptor in the modulation of mood and responses to stress exposure [179,180,181,182,183]. Several pre-clinical studies reported the effectiveness of the NOP blockage in producing anti-depressant-like effects under different experimental conditions [184,185,186,187,188,189,190,191,192]. Among these, a repeated administration of NOP antagonists was able to counteract the depressive-like behaviors induced by exposure to chronic stress [185,191]. The neurobiological mechanisms underlying the antidepressant effects of NOP antagonists are not fully understood. However, they can be at least in part attributed to the ability of N/OFQ to inhibit the release of monoamines in various brain regions. NOP antagonists, by counteracting this inhibitory effect, may lead to an increase in synaptic levels of serotonin and norepinephrine, which could explain their antidepressant action [182]. Additionally, as reviewed in [180], different stress models upregulate the N/OFQ system, and the NOP inhibition during stress exposure is able to reduce the deleterious effects of stress on mood [182,193,194], anxiety levels [195], and memory [196], while the administration of NOP agonists during stress exposure worsens the effects of stress [194,197,198]. Thus, the N/OFQ–NOP system seems to be activated during stress exposure, and its activation likely contributes to the deleterious effects of stress and to maladaptive behaviors possibly leading to psychopathologies. Of note, the release of N/OFQ in the ventral tegmental area of freely moving mice during stress (tail suspension) has been recently demonstrated using the NOPLight sensor [199]. On the other hand, NOP blockage confers resilience to stress exposure, and NOP antagonists may be useful in vulnerable subjects for preventing depressive episodes and stress-triggered diseases. This hypothesis is corroborated by some evidence in humans, showing that NOP receptor is up-regulated in healthy humans after an acute stressful challenge [200] and in women that developed post-traumatic stress disorder (PTSD) after sexual violence [201]. Considering the involvement of the N/OFQ–NOP pathway both in pain transmission and stress response, the involvement of this system in stress-induced pain modulation has been hypothesized. Here we report and discuss the results of the studies that addressed this question, both referring to SIA and to SIH.

## 6. Role of the N/OFQ–NOP Receptor System at the Interplay between Stress and Pain

### 6.1. The N/OFQ–NOP Receptor System and SIA

Pain was the first field of research in which the activity of the N/OFQ–NOP receptor system has been investigated. In the frame of these studies, attention has been paid to SIA, especially considering the role played by classical opioid receptors in this phenomenon and the link between those receptors and the NOP receptor. Different papers reported that N/OFQ injected into the brain counteracts SIA in rodents under different experimental conditions. In a first paper, Suaudeau et al. [202] demonstrated that the i.c.v. injection used to administer N/OFQ into the brain is per se a stressful practice able to induce SIA. Thus, they hypothesized that the pro-nociceptive effect induced by supraspinal N/OFQ may be due at least in part to SIA inhibition. In line with this, N/OFQ (1 nmol, i.c.v.) was able to counteract SIA evoked by 3 min of forced swimming, both in water at 15 °C and at 32 °C [44]. Of note, while SIA elicited by low-severity swim stress (32 °C) is blocked by naloxone, SIA induced by higher-severity swim stress (15 °C) is naloxone-insensitive, suggesting the involvement of endogenous systems different from opioids. The evidence that N/OFQ counteracts both opioid-dependent and opioid-independent SIA suggests that it acts not only modulating the opioidergic transmission but also interfering with other neurotransmitters having a role in SIA. Orexins are reported to be necessary to obtain SIA after 30 min of restraint [203,204]. Interestingly, direct interactions between N/OFQergic and orexinergic neurons in the lateral hypothalamus of mice and rats have been described, and N/OFQ in the hypothalamus inhibited orexinergic transmission [56,205,206]. The administration of orexin-A restored SIA abolished by N/OFQ [204]. Moreover, when microinjection experiments have been performed on rats, the brain area important for N/OFQ effects on SIA resulted in the perifornical area of the lateral hypothalamus, where the orexin neurons are located [56]. This evidence suggests that N/OFQ may inhibit SIA by not only counteracting the activity of the opioid system but also modulating orexin release and effects.

As far as the activation of the endogenous N/OFQ–NOP system during SIA is concerned, this has been investigated using the NOP antagonist [Nphe^1^]N/OFQ(1-13)NH_2_ [44] and mice knockout for the preproN/OFQ gene [207]. In these studies, SIA has been evoked by forced swimming, and both studies demonstrated increased SIA in mice in which the N/OFQ–NOP system has been blocked. In particular, [Nphe^1^]N/OFQ(1-13)NH_2_ increased the opioid-dependent component of SIA, while mice lacking the N/OFQ peptide displayed enhanced SIA, especially after repeated stress exposure, when SIA is almost extinguished in wild-type mice. These results suggest that the endogenous N/OFQ is released during stress exposure and counteracts those mechanisms leading to SIA.

### 6.2. The N/OFQ–NOP Receptor System and SIH

The role of the NOP receptor in stress-induced hyperalgesia has been studied mainly by Zhang and colleagues using a post-traumatic stress disorder model named single-prolonged stress (SPS). This experimental procedure consists of exposing rats to 2 h of complete restraint, followed by 20 min of a forced swim test, anesthesia induction with diethyl ether, and 7 days of isolation. The protocol leads to the development of paw mechanical and thermal allodynia lasting for 28 days post-SPS. SPS significantly increased the N/OFQ levels in cerebrospinal fluid (CSF) and serum [208,209,210], as well as in periaqueductal gray (PAG) [210,211]. The treatment with the NOP antagonist JTC-801 reverted the hyperalgesia induced by SPS, suggesting that the increased endogenous N/OFQ has a role in the maintenance of the hyperalgesia and allodynia produced by stress [211]. This hypothesis has been confirmed by genetic studies that demonstrated that rats knockout for the NOP receptor gene (NOP(−/−)) failed to develop allodynia and hyperalgesia after SPS [212]. Thus, the endogenous N/OFQ increases stress vulnerability not only in terms of psychopathological development (i.e., depression and anxiety) but also in terms of pain sensitivity. The mentioned studies support the investigation of NOP antagonists as preventive agents for those situations in which stress precipitates pain. Anyway, it is worth noting that the studies now available on NOP and SIH suffer from some limitations, i.e., the use of a single stress model, the use of a single NOP antagonist, and the use of a single species. Thus, to firmly propose NOP antagonists as prophylactic treatments for stress-driven pain pathologies, further research is needed using models of stress different from SPS, different NOP antagonists, i.e., SB-612111 [213,214] and the clinically viable BTRX-246040 (also known as LY2040094) [215,216], and NOP(−/−) animals. Interestingly, while male NOP(−/−) rats were protected from SIH, no differences were recorded between female wild-type and NOP(−/−) rats in their liability to SPS-induced SIH [211]. This suggests that a sexual dimorphism may exist in the role played by the N/OFQ–NOP system in stress reactions. Right now, the majority of this research addressing the NOP receptor in relation to stress and/or depressive-like behavior has been performed in male animals, and this is at least in part due to the difficulty in reproducing some of the experimental model in female rodents [184]. However, if the NOP activation during stress exposure produces different actions in male and female mice is a question that deserves attention and needs to be further investigated.

Restraint stress is commonly used to induce visceral pain and mimic irritable bowel syndrome in rodents [217,218]. Under these experimental conditions, the ability of the N/OFQ to revert the colon hypersensitivity has been assessed [219]. It was demonstrated that N/OFQ reduces visceral pain induced by stress only when given in the periphery but not into the brain. This study is in line with several other investigations reporting the analgesic effects of NOP agonists. Anyway, the authors did not test if the NOP blockage during restraint may alter the rat’s liability to develop visceral pain. Thus, to the best of our knowledge, no data are now available to foresee the effectiveness of NOP antagonists in preventing abdominal pain in irritable bowel syndrome patients.

**Figure 1:**
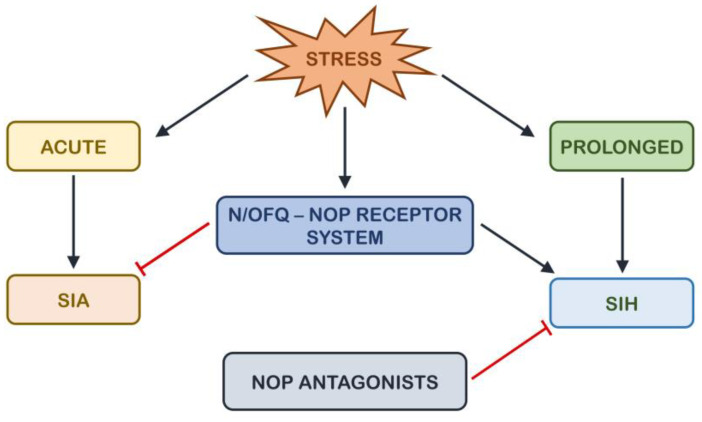
Interactions between stress, pain perception, and the N/OFQ–NOP system. Stimulatory arrows are represented in black, while inhibitory arrows are represented in red.

The mechanisms by which NOP activation worsens the effects of stress, thus facilitating SIH, have not been investigated yet. It is well known that N/OFQ reduces monoamine release in different brain areas and that this is important for the antidepressant effects of NOP antagonists (for a review, see [182]). As stated before, monoamines seem to play a role in SIH, and the potentiation of synaptic levels of serotonin and norepinephrine is associated with SIH reduction [66,70,80,83,128]. Thus, we can speculate that the activation of the N/OFQergic system during stress exposure may foster SIH by counteracting monoaminergic transmission and the analgesic effects of endogenous monoamines. This N/OFQ action is blocked by the administration of NOP antagonists. On the other hand, interactions between the HPA axis and the N/OFQ–NOP system have also been reported (for a review see [180]), and our research group recently demonstrated that the blockade of corticotropin-releasing hormone receptor 1 (CRFR_1_) and glucocorticoid receptor (GR) counteracts the detrimental effects of a NOP agonist given during stress exposure on depressive-like behaviors [197]. Thus, it can be hypothesized that NOP activation facilitates SIH and NOP blockage protects from SIH through the interaction both with the monoaminergic and the HPA systems. This is, however, a mere speculation that needs to be experimentally tested in future studies. Figure 1 illustrates the interactions between stress and pain perception, highlighting our hypothesis that these interactions are modulated by the N/OFQ–NOP system.

## 7. Conclusions and Future Perspectives

SIA and SIH represent two opposite responses to stress, reflecting the complex relationship between stress and pain. SIA is generally considered an advantageous adaptive response to stress. On the contrary, SIH is a deleterious condition that arises when the body fails to adapt to a prolonged stressful environment. To date, rodent models of SIA, SIH, and stress-induced migraine represent the main tools to study these complex phenomena. Despite several authors pointing out that a translational gap exists between rodent models and humans, the utility of the information obtained from rodents is widely recognized. On the other side, the use of animals in research presents ethical concerns that strongly prompt the development of alternative methods (e.g., organoids and three-dimensional culture systems, organ-on-a-chip systems) or the use of species less complex than rodents (e.g., zebrafish, xenopus, drosophila). In the field of pain, to reduce the number of rodents used, invertebrate and lower vertebrate have already been employed [220]. Similarly, the use of zebrafish in stress and psychiatric research has been reported [221]. Thus, there are promising foundations for the development of SIA and SIH models using animals with a simpler nervous system than rodents. This represents an open challenge that should be pursued in the near future to achieve at least a partial replacement. Of note, the complete replacement should still remain the ultimate goal, but scientific limitations make this goal difficult to obtain for this research area at present. A large body of evidence suggests that the N/OFQ-NOP system is active during stress exposure. N/OFQ from one side counteracts SIA; from the other side, it promotes SIH, thus facilitating a maladaptive response to stressful situations. NOP antagonists may promote an active coping strategy to stress and protect vulnerable subjects from stress-driven pain diseases. This intriguing hypothesis is based on limited pre-clinical findings, and further research in the field is clearly needed. Interestingly, some analogies between the NOP and the kappa opioid receptor, as well as the effects of NOP antagonists on stress resilience, suggest the utility of this class of compounds for the prophylaxis of migraine induced by stress. As stated above, animal models of this pathology have been developed and are useful tools to test the therapeutic potential of new candidate drugs. NOP antagonists as well as the phenotype of NOP(−/−) mice or rats are worthy to be investigated using these experimental models. Moreover, women predominately suffer from chronic pain conditions, including those pain conditions sensitive to stress exposure (i.e., migraine, fibromyalgia, and irritable bowel syndrome) [222,223,224,225]. The high female prevalence of several pain diseases suggests that differences may exist across sexes in the biological mechanisms underlining these pathologies and that these differences may apply also to the effects of stress on these conditions. Thus, future studies aimed at investigating the interplay between NOP and SIH should be performed considering sex as an experimental variable. Finally, from a translational point of view, it is worth noting that a NOP antagonist reaching clinical trials is already available (BTRX-246040 [214,215]). In small proof-of-concept clinical studies, BTRX-246040 was safe and well tolerated and showed efficacy in depressed [226] and alcohol dependent [227] patients. Thus, BTRX-246040 may represent a critical tool to confirm or refute our hypothesis, assessing the real effectiveness of NOP antagonists to protect vulnerable individuals from stress-exacerbated pain.

## 8. Limitation of the Review

This is a narrative review providing an overall summary of the main approaches used to induce SIA, SIH, and stress-induced migraine and the evidence linking the N/OFQ–NOP system to SIA and SIH. Consequently, some experimental protocols capable of induced SIA or SIH, but not commonly used, have been omitted, as well as some references that might be missing.

## Figures and Tables

**Table 1:** Summary of the effects produced by the most commonly used stress procedures on pain perception.

Specie	Sex	Experimental Conditions	Outcomes	Comments	References
**Footshock**					
*Stress induced analgesia*					
Rat	Male	90 s–3 min, 1.6 mA	Thermal analgesia		[27,32]
		20 min, 1.6 mA, intermittent	Thermal analgesia		[27,28]
Mouse	Male	3–30 min, 0.6 mA	Thermal analgesia		[20,25]
**Forced swim**					
*Stress induced analgesia*					
Rat	Male	3–6 min, 25 °C	Thermal analgesia		[14,47,48,50]
			Reduced nociceptive behaviors in the formalin test		[22,23,24,34,35,36,37,49]
		3.5 min, 2 °C	Thermal analgesia		[38]
Mouse	Male	2–3 min, 32 °C	Thermal analgesia		[18,33,40,44]
		2–3 min, 15 °C	Thermal analgesia		[18,44]
		2 min, 2 °C	Thermal analgesia		[40]
	Male and female	3 min, 32 °C	Thermal analgesia	Higher SIA in female mice; naloxone completely blocked SIA in male mice but only partially in female mice	[42]
		3 min, 15 °C	Thermal analgesia	Different sensitivity between male and female mice to NMDA receptor antagonists	[46]
	Female	3 min, 32 °C	Thermal analgesia		
*Stress induced hyperalgesia*					
Rat	Male	(10–20 min) × 3 days, 24–26 °C	Thermal and mechanical hyperalgesia		[65,66,68,69]
			Increased nociceptive behaviors in the formalin test		[66,70,71,72]
			Increased CFA-induced mechanical and thermal hyperalgesia		[73,74,76,77]
		(10–20 min) × 10 days, 24–26 °C	Increased nociceptive behaviors in the formalin test	SIH obtained in Sprague-Dawley but not in Wistar Kyoto rats	[78]
	Female	(10–20 min) × 3 days, 24–26 °C	Thermal and mechanical hyperalgesia		[67]
			Increased CFA-induced mechanical and thermal hyperalgesia		[75]
**Restraint**					
*Stress induced analgesia*					
Rat	Male	30 min–3 h	Thermal analgesia		[15,53,56,58,64]
			Reduced nociceptive behaviors in the formalin test		[54,55,59,60,61,63]
	Male and female	1 h	Thermal analgesia	No major differences between male and female	[51]
Mouse	Male	30 min–3 h	Thermal analgesia		[17,19,62]
*Stress induced hyperalgesia*					
Rat	Male	(30 min) × 3 days	Increased PGE_2_-induced mechanical hyperalgesia		[105]
		(1 h) × 35–70 days	Thermal and mechanical hyperalgesia	No effects in the Randal and Selitto test and in the tail withdrawal test at different temperature [88]	[86,87,90,111]
			Increased nociceptive behaviors in the formalin test		[83,104]
		(2 h) × 14–28 days	Increased bee venom-induced nociceptive behaviors		[106]
		(6 h) × 14–21 days	Mechanical hyperalgesia		[89,91,98,101,102,103]
	Male and female	(1 h) × 40 days	Increased nociceptive behaviors in the formalin test	SIH only in male rats	[51]
		(2 h) × 14–28 days	Thermal and mechanical hyperalgesia	No major differences between male and female in SIH; when stress was applied with infraorbital nerve chronic constriction injury, females displayed higher hyperalgesia [107]	[88,93,106,107]
Mouse	Male	(3 h) × 10 days	Thermal and mechanical hyperalgesia		[96]
		(4 h) × 10 days	Thermal and chemical corneal hyperalgesia		[110]
		(6 h) × 7–28 days	Thermal and mechanical hyperalgesia		[84,95,97,100]
			Increased low-back pain signs induced by NGF	Vertical restraint	[108]
	Male and female	(2 h) × 28 days	Mechanical hyperalgesia	No major differences between male and female	[93]
		(6 h) × 28 days	Thermal and mechanical hyperalgesia	No major differences between male and female	[92,94]
	Female	(2 h) × 28 days	Increased nociceptive behaviors in an endometriosis model		[109]
*Stress induced migraine*					
Mouse	Male	(1 h) × 3 days	Periorbital mechanical allodynia that disappeared after 14 days, sensitization to a subthreshold dose of migraine-inducing drug		
	Male and female	(2 h) × 3 days	Periorbital mechanical allodynia that disappeared after 14 days, sensitization to a subthreshold dose of migraine-inducing drug	No major differences between male and female	[137,138]
**Repeated cold**					
*Stress induced hyperalgesia*					
Rat	Male	(night: 4 °C; day: 24 °C and 4 °C switched every 30 min) × 5 days	Thermal and mechanical hyperalgesia, hyperalgesia in the Randal and Selitto test		[120,121,122,123,125]
Mouse	Male	(night: 4 °C; day: 24 °C and 4 °C switched every 30 min) × 3–7 days	Thermal and mechanical hyperalgesia		[117,118,119,124]

CFA, complete Freund’s adjuvant; NGF, nerve growth factor; NMDA, N-methyl-D-aspartate; PGE_2_, prostaglandin E_2_; SIA, stress-induced analgesia; SIH, stress-induced hyperalgesia.
